# The Use of Hall's Technique Preformed Metal Crown (HTPMC) by Pediatric Dentists in Malaysia

**DOI:** 10.1155/2021/8424206

**Published:** 2021-12-22

**Authors:** Fabiha Jesmin, Aimi Kamarudin, Fadzlinda Baharin, Wan Muhamad Amir Bin W. Ahmad, Mahmud Mohammed, Anand Marya, Pietro Messina, Giuseppe Alessandro Scardina, Mohmed Isaqali Karobari

**Affiliations:** ^1^Department of Paediatric Dentistry, Delta Medical College-Dental Unit, Mirpur, Dhaka, Bangladesh; ^2^Paediatric Dentistry Unit, School of Dental Sciences (PPSG), Universiti Sains Malaysia, Kota Bharu, Health Campus, Kelantan, Malaysia; ^3^Biostatics Unit, School of Dental Sciences (PPSG), Universiti Sains Malaysia (USM), Health Campus, Kelantan, Malaysia; ^4^Department of Conservative Dentistry & Endodontics, Delta Medical College-Dental Unit, Mirpur, Dhaka, Bangladesh; ^5^Department of Orthodontics, Saveetha Dental College, Saveetha Instititute of Medical and Technical Sciences, Chennai, India; ^6^Department of Surgical, Oncological and Stomatological Disciplines, University of Palermo, Italy; ^7^Department of Restorative Dentistry & Endodontics, Faculty of Dentistry, University of Puthisastra, Phnom Penh 12211, Cambodia; ^8^Conservative Dentistry Unit, School of Dental Sciences, Universiti Sains Malaysia, Health Campus, Kubang Kerian, 16150 Kelantan, Malaysia

## Abstract

**Introduction:**

Hall's technique preformed metal crown (HTPMC) has been used widely by pediatric dentists in developed countries as a new approach for managing decayed primary molars without local anesthesia, caries removal, and tooth preparation. Currently, inadequate information is available regarding the implementation of this technique (HTPMC) in Malaysia. This study is aimed at evaluating the implementation of HTPMC by Malaysia's pediatric dentists and identify the co-occurrence frequencies of the HTPMC implementation with the respondents' demographic profile.

**Materials and Methods:**

A cross-sectional questionnaire-based research was conducted among 65 pediatric dentists in Malaysia. Online questionnaires were distributed to the pediatric dentists employed at public hospitals (MOH) and universities in Malaysia.

**Result:**

It was found that over half of the respondents (65.6%) employed HTPMC. The analysis of the co-occurrence network frequency revealed that a high frequency of female pediatric dentists who were within the age group of 31-40 years old had fulfilled their postgraduation overseas and was employed in the university mainly applied HTPMC.

**Conclusion:**

The application of HTPMC among respondent pediatric dentists in Malaysia was high. However, most respondents considered HTPMC a treatment option only to manage carious primary molar rather than a treatment of choice.

## 1. Introduction

Hall's technique preformed metal crowns (HTPMC) are used widely in developed countries, including the United Kingdom, United States, and Germany [1]. An increase in the application of HTPMC takes place worldwide despite past controversies regarding its success rate. Although many studies have demonstrated its high success rate in managing primary carious teeth, the resistance to using HTPMC remains among pediatric dentists [2]. This resistance among some practitioners is primarily due to complex technical and aesthetic concerns [2]. In 1997, a general dental practitioner, Dr. Norna Hall from North Scotland, applied an unconventional method to treat the carious primary molars with a preformed metal crown. In this case, a preformed metal crown was used without administrating local anesthesia, removing caries, or preparing the tooth [1]. Conventionally, preformed metal crowns are placed on primary molars after local anesthesia and crown preparation in young children.

In contrast, provided that HTPMC requires minimum intervention for managing carious primary molars, this technique could be used on especially young children. HTPMC effectiveness is based on the sealing of carious tooth with a preformed metal crown, thus preventing microorganisms from their origin of nutrition, dietary carbohydrate, and the advancement of caries [3, 4]. Besides, a systematic review has demonstrated that the avoidance of caries removal could prevent pulpal exposure [5].

Based on a comparison by Santamaria et al.'s (2018) study comparison between HTPMC and the conventional methods, it was found that HTPMC showed a better outcome and more extended longevity compared to the conventional restorations [6]. It has been proven that the HTPMC shows successful management of dental caries in a primary molar. In contrast, another research has demonstrated that GDP, the patients, and their parents preferred HPTMC compared to the conventional restorative approaches for managing primary carious molar [7]. A recent study was performed in Germany to assess the implementation and perspectives of the preformed metal crown by pediatric dentists. It was found that 77% of the respondents did not have familiarity with HTPMC, while 66% of the respondents did not employ HTPMC as this technique was found to be challenging and aesthetic [6].

Malaysian pediatric dentists have been practicing HTPMC in the management of primary carious molar. However, the information regarding the implementation of HTPMC in Malaysia is inadequate. Following a literature review, it was seen that no research had been carried out on the use of HTPMC in Malaysia. This study is aimed at examining the generalizability of the implementation of HTPMC by pediatric dentists in Malaysia. This study would assist in identifying the use rate and the acceptability of HTPMC among dentists, as mentioned above.

## 2. Materials and Method

### 2.1. Ethical Consideration

The research was approved by the responsible committee on human research and ethics, with the research protocol code of USM/JEPeM/19010099. Informed consent was acquired from all the parties involved in the research. Given that the information and responses were regarded as fully confidential and anonymous, the research team members were the only parties allowed to access the information.

### 2.2. Subjects

A total of 45 pediatric dentists offered services under the Ministry of Health (MOH)/Kementerian Kesihatan Malaysia (KKM). On the other hand, the Malaysian Dental Council retained 33 pediatric dentists from private and government universities. It was predicted that the pediatric dentists registered under the hospitals and universities in Malaysia would amount to 78. After incorporating 10% of the nonresponse rate, the purposive selection was performed on 65 pediatric dentists from Malaysia's hospitals and universities to evaluate the application of HTPMC.

### 2.3. Sample Size Calculation

A single proportion formula was applied to compute the sample size to identify the prevalence of HTPMC usage among 78 pediatric dentists in Malaysia. With a predicted prevalence of 96%, the population of pediatric dentists who employed HTPMC as an option of treatment to manage caries in the primary molar was according to Roberts et al.'s (2018) research.

(1) $$\begin{array}{c} n={\left(\frac{Z}{∆}\right)}^{2}p\left(1-p\right), \end{array}$$

where n is the sample required, Z is the normal standard deviation corresponding to 95%confidence interval = 1.96, ∆ is the research precision, which would be 5% of this research as per the recommendation by WHO guidelines for oral health surveys, and *P* is the population proportion needed according to past research findings.

Following past research findings, 96% of pediatric dentists were employing HPTMC as an option of treatment to manage caries in primary molars [8]. Therefore, the precision of 0.05 at 95% of the confidence interval the total sample size (*n*) was as follows:

(2) $$\begin{array}{c} n=0.96\;\left(1-0.96\right)\;{\left(1.96/0.05\right)}^{2=5}9. \end{array}$$

The sample size with a precision value of 0.05 amounted to 59. Taking into account the 10% nonresponse rate, the final sample size amounted to 65.

### 2.4. Study Design

This cross-sectional research was conducted among the pediatric dentists in Malaysia who work under hospitals and universities. An online survey questionnaire, which comprised 16 close-ended questions, was adopted from the previous research by Roberts et al. 2018 [8]. Pretest and modification of the questionnaire were conducted accordingly. The questionnaire consisted of three sections: demographic characteristics, the use of HTPMCs, and the last section contained questions for the pediatric dentists to answer.

#### 2.4.1. Demographic Section

This sociodemographic section presented the participants' characteristics, including age, gender, province, education history, the clinical setting the respondents were employed in, job title, and the years of their practice as pediatric dentists.

#### 2.4.2. Usage of HTPMC

The second segment highlighted the questions regarding HTPMC use, which addressed whether this approach was the preferred treatment modality, treatment methods for various categories of carious lesions, and the duration for using the approach. They were also asked whether they would place HTPMC under inhalation sedation and general anesthesia for special-needs children. A question was also included on whether the respondents agreed that HTPMC was suitable to be used by undergraduate students, postgraduate students, dental officers, and postbasic staff nurses.

#### 2.4.3. Perceived Barriers

In the last section, a single question is provided that represents the perceived barriers during the use of Hall's technique preformed metal crown. This is a continuation of the first question in the second section. In this section, they need to mention the barriers that could cause the reluctance to use HTPMC.

### 2.5. Data Collection

Data collection began with distributing the data collection sheet (conversion of the sheet into an online survey) to the respondents through the email chosen based on inclusive and exclusive standards. Anonymity was maintained for the entire questionnaires employed in this research, while data entry into the SPSS software was performed. Furthermore, the research team members were the sole parties accessing the data, while data was displayed in grouped data and would not classify the respondents as individuals. Notably, ethical approval from National Medical Research, Malaysia (NMR), was required for respondents from MOH and Malaysia's universities. In this case, data collection would be performed through questionnaire distribution if the respondents did not present a response within 30 days. The respondents were called to be reminded of this matter. A cover letter was included in the survey to elaborate on the purpose of the data collection and the questionnaire distribution to all pediatric dentists in Malaysia.

## 3. Data Entry and Statistical Analysis

Data entry and statistical analysis were performed using SPSS version 24.0. Descriptive analysis was conducted, including the distribution of percentage for demographic profiles and questions associated with HTPMC. Multiple response questions were scored following the frequency of the chosen responses. This was followed by calculation of the frequencies and interpretation of results. Moreover, SPSS software was employed to analyze the co-occurrence frequencies.

## 4. Result

The response rate in this study amounted to 49%, representing 32 out of 65 respondents who submitted the responses online.

### 4.1. Demographics

Most of the respondents (50%) were aged 31 to 40 years old, while nine respondents (28.1%) were aged 41 to 50 years old. The majority of the respondents, which amounted to 27 (84.4%), were presented by female pediatric dentists. Furthermore, 14 respondents (43.8%) served in hospitals that have been registered under the Ministry of Health (MOH), while 18 respondents (56.3%) served in private and government universities. Eleven respondents (34.4%) served as lecturers in universities, nine respondents (28.1%) were employed as specialists, and five respondents (15.6%) served as consultants in Malaysia's hospitals. Nine respondents (28.1%) worked for over 20 years, while eight (25%) gained 11-15 years of work experience in practice. Following that, 24 respondents (77.4%), the majority, had fulfilled postgraduation overseas, while seven respondents (22.6%) had fulfilled postgraduation from Malaysia.

### 4.2. Usage of HTPMC

21 out of 32 pediatric dentists are using HTPMC in their regular practice, which indicated the frequent use of HTPMC among the respondent pediatric dentists in Malaysia in their regular practice. However, this result did not apply to all pediatric dentists in Malaysia. Meanwhile, 14 respondents (66.7%) mentioned using HTPMC as a treatment option for managing a carious primary molar. On the other hand, four respondents (19%) want to use HTPMC as a treatment of choice for managing carious primary molar, and seven respondents (33.3%) want to use HTPMC upon facing failure in managing the carious tooth with the conventional approach. Generally, treatment of choice refers to choosing HTPMC as a primary treatment for managing carious primary molar over the conventional restorative methods, whereas treatment option means they only choose HTPMC as an alternative or secondary treatment when conventional treatment with the bur was impossible.

Among the 21 respondents, 10 (47.6%) mentioned that they would prefer to apply HTPMC in cavitated interproximal carious teeth, while four respondents (19.0%) mentioned using HTPMC in cavitated occlusal carious teeth. However, five respondents (23.8%) did not prefer the application of HTPMC for noncavitated occlusal carious teeth, while two respondents (9.5%) did not wish to employ it for noncavitated interproximal carious teeth (Table 1). Following that, Table 2 demonstrates that most respondents (20 respondents, 95.2%) would like to use HTPMC for special needs children. However, 16 respondents (76.2%) preferred fitting the metal crown under inhalation sedation, while five respondents (23.8%) wished to apply it under general anesthesia.

From the answers by 19 pediatric dentists (95%), it was perceived that HTPMC is suitable for postgraduation, while 12 (60%) agreed that it is acceptable for the undergraduate curriculum. It was observed that dental officers 95% (19) and postbasic staff nurses 40% (8) could be trained using HTPMC.  Table 3 illustrates that 44.4% of the respondents did not wish to use the separators or obtain X-Rays when HTPMC was used.

### 4.3. Co-Occurrence Frequency Analysis

**Figure 1:**
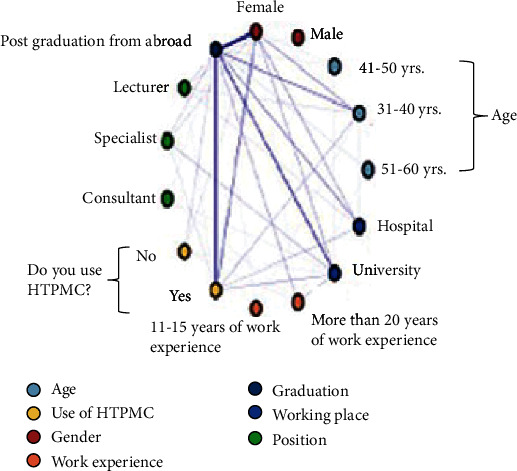
Co-occurrence frequency network between the use of HTPMC with the demographic profile of the respondents.

The co-occurrence frequencies of HTPMC and demographic profile demonstrated a high-frequency network (indicated by dark line) between respondent female pediatric dentists aged from 31 to 40 years old, fulfilled their postgraduation overseas, and applied HTPMC upon work in the university (Figure 1).

## 5. Discussion

In this study, the sample size consisted of 65 pediatric specialists, and a low response rate of 49% was recorded. However, 41% and 38.9% response rates were recorded in past questionnaire-based research studies on using HTPMC [6, 8]. Therefore, the response rate from this study was comparatively higher. A study by Cunningham et al. (2015) highlighted that 35% of the response rate is comparable for online surveys to eliminate the issue of response bias [9]. For the current study, the response rate mentioned earlier was 49%, which seems low and could have been higher had we met the pediatric dentists personally for data collection. The sample size of the present survey was restricted to pediatric dentists employed in universities, public hospitals, and private clinic practitioners excluded from the survey. Several research works were performed among the general dental practitioners and private clinic practitioners (Dean et al., 2011; John, 2016; Santamaría et al., 2018), while this study involved a sample size of 65. The predicted number of registered pediatric dentists under universities and public hospitals in Malaysia was 78, which was adequate for conducting this research by omitting private practitioners.

The study results suggested that there was high usage of HTPMC amongst the respondent pediatric dentists in Malaysia. However, most of them would apply HTPMC to manage carious primary molars instead of the chosen treatment or primary treatment option. This research highlighted that several practitioners perceived the “second-best” feature about the HTPMC, which is applied only when other techniques are not applicable. The online survey performed by Hussein et al. (2020) to evaluate global pediatric dentist opinions/use of HTPMC was only used by over half of those respondents. Conventional restorations remained the preferred option even among HT users [10]. On the other hand, in a recently conducted systematic review, the authors mentioned that HT remained the preferred option among the general dentist. Moreover, the study also mentioned that the questionnaire-based study showed better acceptance than face scale-based evaluation [11].

Approval was made by specialists on the general use of HTPMC, with most of them suggesting the training for dental officers. Furthermore, half of the specialists highlighted the importance of HTPMC training among the undergraduate and postgraduate students for the early exposure of HTPMC. Given that HTPMC is a noninterventional treatment, convenient to conduct, and practical, few pediatric dentists recommended training primary staff nurses on HTPMC. It was believed that the use of HTPMC would encourage more practice and could be a favorable treatment option for the management of carious primary molars.

It was found that the use of HTPMC in cavitated lesions and interproximal lesions is more frequent based on Hall's technique manual, which suggests the use of this technique in the presence of two surface lesions or extensive one surface lesions. Contrary to the manual, which recommends partial caries removal and sealant for noncavitated occlusal cavities, including sealant only for cavitated occlusal cavities, a notable amount of respondents employed HTPMC for noncavitated and cavitated occlusal cavities [8]. This condition could be attributed to the convenience of fitting HTPMC in comparison to partial caries removal. A majority of the children in Malaysia are classified as high caries risk; thus, the placement of HTPMC for noncavitated and cavitated occlusal cavities is more reasonable for preventing sealant failure than sealants.

A majority of the children arranged for oral rehabilitation under general anesthesia (GA) were recorded with negative behavior towards dental treatment, which led to challenging extraction of radiographs during the arrangement for treatment [12]. Based on Hall's technique manual, a thorough assessment that includes a radiograph must be performed to omit the irreversible pulpal involvement before the Hall crown is placed. It was observed from this research that the respondents were not willing to employ HTPMC under the GA setting; although, they were highly accepting of the success rate of HTPMC. The absence of radiographs could cause this condition during GA.

Malaysia's Ministry of Health currently promotes oral healthcare services for special needs children [13]. Given that these children belong to the group with high caries risk, they require a durable restorative material to restore carious teeth. Furthermore, most special-needs children display challenging behavior, which was a factor for the majority of the respondents (95.2%) in this research to employ HTPMC for special needs children due to the convenience in conducting HTPMC and its remarkable longevity. In a recent study on European postgraduate pediatric dentistry students, HTPMC was chosen as an option more often for anxious children than children who were not anxious, i.e., not as the treatment of choice for nonanxious children [14].

Notably, HTPMC is a treatment option for anxious children with a high success rate, suggesting that future specialists are more pragmatic in employing this method. However, it has been observed that a low number of specialists (34.4%) remain reluctant to use HTPMC as they do not prefer to fit the crown without the removal of caries. In a questionnaire-based study performed in Scotland, it was found that 48% of respondents employed HTPMC, while the respondents who did not do so had a preference for further training [15]. Despite identifying the effectiveness of HTPMC compared to conventional restorations, the factors of specialists' refusal to use it could not be determined. The current systematic review of Jesmin et al. (2020) demonstrated that HTPMC led to responses ranging from “no discomfort” to “mild discomfort” compared to conventional restorations [16].

According to the co-occurrence frequency analysis, an established relationship was present between the demographic profile of the respondents upon the application of HTPMC. The frequency network (Figure 1) demonstrated that the respondent female pediatric dentists aged from 31 to 40 years old, having fulfilled their postgraduation overseas and worked in the university, showed more frequent use of HTPMC. Moreover, the respondents of this research, who had fulfilled their postgraduation from Europe, showed confidence regarding this technique. Besides, they reported an equal use of HTPMC in their routine practice.

In a recent study, the author mentioned that most pediatric dentists across the globe do not use HT. Moreover, only half of the pediatric dentists who participated in that study used HT for treating carious primary molar [10]. The current study result has shown a similar output pattern; the Malaysian pediatric dentists showed reluctance to use HTPMC to treat carious molar after having enough evidence of its success rate.

Previously, Midani et al., a retrospective study was performed to assess the success rate of standard HT and modified HT. The modified HT involved proximal tooth slicing, allowing the PMC to fit without separation and providing minimal occlusal cusps reduction without caries removal and local anesthesia. The study result showed that comparing crowns performed with no tooth preparation to crowns performed with proximal slicing, and no differences were observed [17]. Identifiable barriers such as lack of training, substandard dentistry, and perceived lack of evidence reduced its use. Therefore, it was believed that the HTPMC is not used primarily due to inadequate training or confidence in the available evidence.

### 5.1. Limitations

As explained in the discussion, the response rate for this study was 49%, which could have been improved if the data collection had been done physically instead of using an online survey. Also, in this case, online data collection was the only feasible method for data collection, considering the current situation. It has been seen that health professionals usually offer a low number of responses to online surveys ^15^. Moreover, the dentists who responded to the survey are more likely to be interested in HTPMCs than those who did not respond. In addition to that, we can assume that the nonrespondent of this study may have less interest in using HPTMC for managing carious primary molars. The nonrespondent bias may affect the overall outcome of the current study.

### 5.2. Recommendations

For future studies, general dental practitioners in Malaysia can be included in the sample to assess the knowledge, attitude, and practices regarding HPTMC usage among Malaysian dentists. The data obtained from this study can also be utilized for carrying out multicultural studies to analyze differences in HTPMC usage across different countries.

## 6. Conclusion

A notable result was found in this study that the respondent pediatric dentists in Malaysia preferred HTPMC. In addition to that, from the demographic assessment, we found that the respondents using HTPMC to manage carious premolars are mostly aged from 31 to 40 years old, having fulfilled their postgraduation overseas and working under the university.

## Figures and Tables

**Table 1:** Frequency of using HTPMC in a given situation.

	*a*	*b*	*c*	*d*
Never	23.8% (5)	0%	9.5% (2)	0%
Rarely	47.6% (10)	19.0% (4)	19.0% (4)	0%
Sometimes	28.6% (6)	62.0% (13)	62.0% (13)	52.4% (11)
Always	0%	19.0% (4)	9.5% (2)	47.6% (10)
Total	100% (21)	100% (21)	100% (21)	100% (21)

a: the use of HTPMC in noncavitated occlusal carious teeth; b: the use of HTPMC in cavitated occlusal carious teeth; c: the use of HTPMC in noncavitated interproximal carious teeth; d: the use of HTPMC in cavitated interproximal carious teeth.

**Table 2:** The situation for the use of HTPMC.

	Special need children	Under inhalation sedation	Under general anesthesia
Yes	95.2% (20)	76.2% (16)	23.8% (5)
No	4.8% (1)	23.8% (5)	76.2% (16)

**Table 3:** Frequency of pediatric dentists who do not use the following steps for HTPMC placement.

Protocol		
Variable	Responses	
	N	Percentage
Take consent from parents (verbal or written)	1	11.1%
Placement of separators	4	44.4%
X-rays (OPG, bitewing, PA view)	4	44.4%
Total	9	100.0%

## Data Availability

Any data used in the current article can be provided upon request from the corresponding author.

## Supplementary Materials

Supplementary Materials The Supplementary file consists of the questionnaire used in the current study.
