# Supplementary material for: The Use of Hall's Technique Preformed Metal Crown (HTPMC) by Pediatric Dentists in Malaysia
Source: Biomed Res Int. 2021 Dec 22;2021:8424206. doi: 10.1155/2021/8424206 (PMC8716193; doi:10.1155/2021/8424206)
Supplement: Supplementary Materials — The Supplementary file consists of the questionnaire used in the current study. [file 8424206.f1.docx]

**
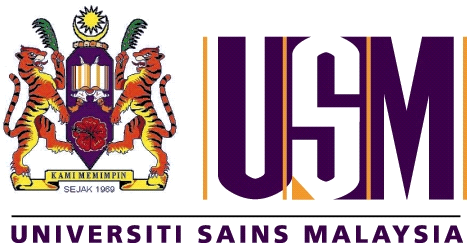
**

**Dear all,**

I am currently conducting a research on ‘**The use of Hall Technique by Paediatric Dental Specialists in Malaysia’** for my MSc in Universiti Sains Malaysia (USM). This study was approved by **JEPeM, USM**. Study protocol code is **USM/JEPeM/19010099**. We appreciate your feedback and responses to the questionnaire attached.

There is **no right or wrong answer** as this instrument is generated for **academic purposes only.** We would be grateful if you could provide responses that truly reflected your own opinion. We can assure you that all information and responses that you provide will be treated as **strictly confidential & anonymous.**

Your cooperation is highly appreciated and valuable. Should you have any quarry regarding to this survey please do not hesitate to contact at +601111001428 or email at [fabihajesmin@student.usm.my](mailto:fabihajesmin@student.usm.my). Your truthful participation is truly appreciated.

Thank you.

Regards,

**Dr. Fabiha Jesmin**, Postgraduate Student at School of Dental Sciences, Universiti Sains Malaysia (USM)

**Dr. Aimi Binti Kamarudin** as Main Supervisor, **Dr. Fadzlinda Baharin** and **Dr. Wan Muhammad Amir Bin Wan Ahmad** as Co-supervisor at School of Dental Sciences, USM

| ***Section1 (Demographic Profile)***  ***Fill in the blank and (√ ) tick all applicable boxes*** |
| --- |

| **Gender**:   \| Male \|  \| \| --- \| --- \| \| Female \|  \|   **Age**:   \| 30 or younger \|  \| \| --- \| --- \| \| 31 to 40 \|  \| \| 41 to 50 \|  \| \| 51 to 60 \|  \| \| 60 or older \|  \|   **State:**   \|  \| \| --- \| | **Institution of Graduation:**   \|  \| \| --- \|   **Institution of Post-graduation:** *(if any)*   \|  \| \| --- \|   **Working experience:**   \| Less than 6 years \|  \| \| --- \| --- \| \| 6 – 10 years \|  \| \| 11 – 15 years \|  \| \| 16 – 20 years \|  \| \| More than 20 years \|  \| | **Position:**   \| Lecturer \|  \| \| --- \| --- \| \| Senior Lecturer \|  \| \| Associate Professor \|  \| \| Professor \|  \| \| Specialist \|  \| \| Consultant \|  \|   **Name of Working place:**   \| Hospital \|  \| \| --- \| --- \| \| University \|  \| |
| --- | --- | --- | --- | --- | --- | --- | --- | --- | --- | --- | --- | --- | --- | --- | --- | --- | --- | --- | --- | --- | --- | --- | --- | --- | --- | --- | --- | --- | --- | --- | --- | --- | --- | --- | --- | --- | --- | --- | --- | --- | --- | --- | --- | --- | --- |

| ***Section 2***  ***Fill in the blanks and (√ ) tick all applicable boxes*** |
| --- |

|  | ***Yes*** | ***No*** |
| --- | --- | --- |
| **Do you use Hall’s Technique?**  *(if no please complete section 3 only)* | **1** | **2** |

| **How long you have been using Hall’s Technique?** |  |
| --- | --- |
| Over 5 years |  |
| 4 years |  |
| 3 years |  |
| 2 years |  |
| 1 year or less |  |

| **When do you plan or use this technique?** | ***Never*** | ***Rarely*** | ***Sometimes*** | ***Always*** |
| --- | --- | --- | --- | --- |
| Treatment option for carious primary molar | **0** | **1** | **2** | **3** |
| Treatment choice for carious primary molar | **0** | **1** | **2** | **3** |
| Only when unable to use a conventional restoration in a carious primary molar | **0** | **1** | **2** | **3** |

|  | **Use of Hall’s Technique** | ***Never*** | ***Rarely*** | ***Sometimes*** | ***Always*** |
| --- | --- | --- | --- | --- | --- |
| 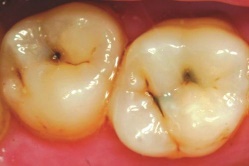 | Would you plan to use Hall Technique in non-cavitated occlusal carious teeth? | **0** | **1** | **2** | **3** |
| 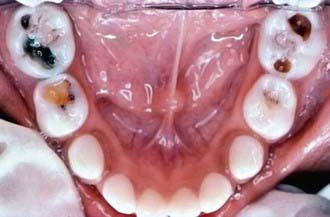 | Would you plan to use Hall Technique in cavitated occlusal carious teeth? | **0** | **1** | **2** | **3** |
| 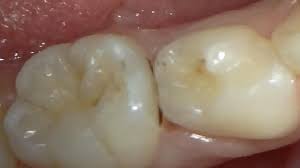 | Would you plan to use Hall Technique in non-cavitated inter-proximal carious teeth? | **0** | **1** | **2** | **3** |
| 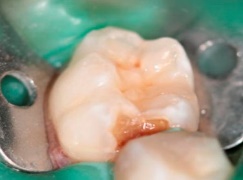 | Would you plan to use Hall Technique in cavitated inter-proximal carious teeth? | **0** | **1** | **2** | **3** |

| **Would you like to use Hall’s Technique in following situation?** | ***Yes*** | ***No*** |
| --- | --- | --- |
| Special need children  *(e.g. Cerebral palsy, downs syndrome, handicapped, autistic)* | **1** | **2** |
| Under Inhalation Sedation | **1** | **2** |
| Under General Anesthesia | **1** | **2** |

| **During Hall’s Technique would you do any of the followings** | **Yes** | **No** |
| --- | --- | --- |
| Take consent from parents (verbal or written) | **1** | **2** |
| Placement of separators | **1** | **2** |
| X-Rays (OPG, Bitewing, PA view) | **1** | **2** |

| **Would you like teach Hall’s Technique any of the following?** | ***Yes*** | ***No*** |
| --- | --- | --- |
| Under graduate dental students | **1** | **2** |
| Postgraduate students | **1** | **2** |
| Dental Officer | **1** | **2** |
| Post basic staff nurse | **1** | **2** |

| ***Section 3***  ***Fill in the blanks and (√ ) tick all the applicable boxes*** |
| --- |

| **Perceived barriers during the use Hall’s technique** | ***Yes*** | ***No*** |
| --- | --- | --- |
| Unable to find the right size of the crown | **1** | **2** |
| Having problem with placing separators | **1** | **2** |
| Don’t like to place the metal crown with GIC without removing caries | **1** | **2** |
| Due to the cost of metal crown | **1** | **2** |
| Don’t feel confident about this technique | **1** | **2** |
| Other (state it) | | |

***Thank you for your cooperation***
